# Mucin CYS domain stiffens the mucus gel hindering bacteria and spermatozoa

**DOI:** 10.1038/s41598-019-53547-x

**Published:** 2019-11-18

**Authors:** Bastien Demouveaux, Valérie Gouyer, Catherine Robbe-Masselot, Frédéric Gottrand, Tetsuharu Narita, Jean-Luc Desseyn

**Affiliations:** 1 Inserm, Univ. Lille, CHU Lille, LIRIC UMR 995, F-59000 Lille, France; 2 CNRS, Univ. Lille, UMR 8576, Unité de Glycobiologie Structurale et Fonctionnelle (UGSF), F-59000 Lille, France; 3 CNRS, PSL Research University, UPMC Univ. Paris 06, ESPCI Paris, UMR 7615, Laboratoire Sciences et Ingénierie de la Matière Molle, 10 rue Vauquelin, 75231 Paris, Cedex 05 France; 4 Global Station for Soft Matter, Global Institution for Collaborative Research and Education, Hokkaido University, Sapporo, Japan

**Keywords:** Biopolymers in vivo, Glycobiology

## Abstract

Mucus is the first biological barrier encountered by particles and pathogenic bacteria at the surface of secretory epithelia. The viscoelasticity of mucus is governed in part by low energy interactions that are difficult to assess. The CYS domain is a good candidate to support low energy interactions between GFMs and/or mucus constituents. Our aim was to stiffen the mucus from HT29-MTX cell cocultures and the colon of mice through the delivery of a recombinant protein made of hydrophobic CYS domains and found in multiple copies in polymeric mucins. The ability of the delivery of a poly-CYS molecule to stiffen mucus gels was assessed by probing cellular motility and particle diffusion. We demonstrated that poly-CYS enrichment decreases mucus permeability and hinders displacement of pathogenic flagellated bacteria and spermatozoa. Particle tracking microrheology showed a decrease of mucus diffusivity. The empirical obstruction scaling model evidenced a decrease of mesh size for mouse mucus enriched with poly-CYS molecules. Our data bring evidence that enrichment with a protein made of CYS domains stiffens the mucin network to provide a more impermeable and protective mucus barrier than mucus without such enrichment.

## Introduction

Mucus is a complex biological fluid that forms a viscoelastic barrier, which protects, hydrates, and lubricates the secretory mucosa^1^. Mucus plays a crucial role in innate immunity by forming a semipermeable gel, which houses bacteria and viruses far from the epithelium^2,3^. There is a strong interplay between mucus structure, viscoelasticity, and permeability as illustrated in the cervix where the change of mucus ultrastructure throughout the ovulatory cycle allows spermatozoa passage only during the ovulatory phase and limits the passage of the vaginal microbiota to the uterine cavity during pregnancy^4,5^. Mucus gels are highly conserved between species and the epithelial tissues that they cover. However, their functions differ, depending on the tissue and their pathophysiological state. The physical properties of mucus are therefore tightly regulated^6^. Investigation of the properties of native mucus is challenging while commercial lyophilized mucins no longer replicate its physiological properties^7^.

**Figure 1:**
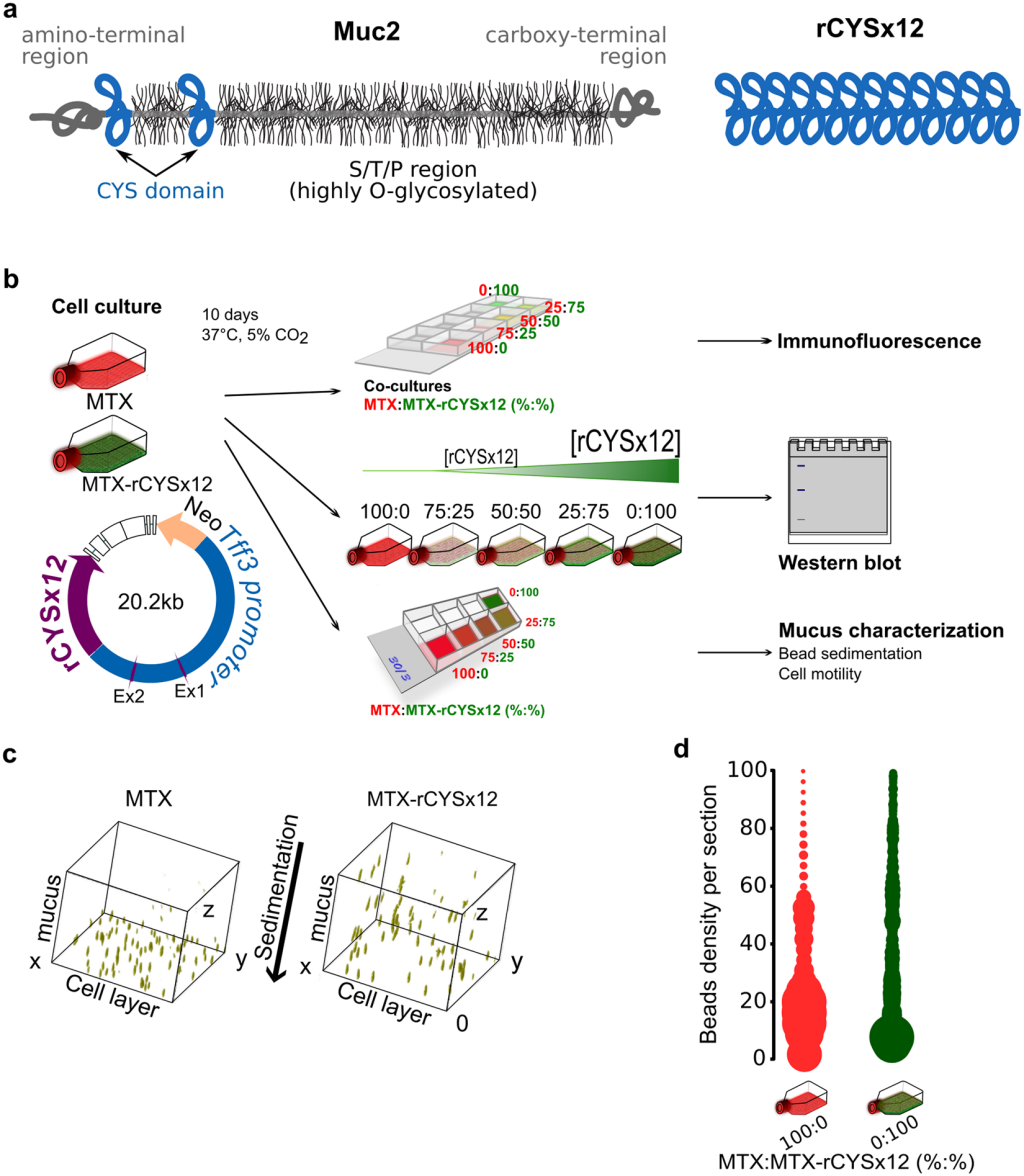
Mucus permeability in MTX:MTX-rCYSx12 cocultures. (**a**) Schematic representation of a monomer of the intestinal mucin Muc2 and the recombinant protein rCYSx12 (not to scale). The mucin CYS domain is depicted in blue and the S/T/P repeats carrying the numerous *O*-glycans are in grey. The recombinant protein rCYSx12 is made of 12 consecutive CYS domains. (**b**) Study design to obtain and to characterize mucus enriched in rCYSx12 from cell culture. MTX cells were transfected with a 20.2-kb vector carrying the first two exons, first intron, and 5 part of intron No. 2 of mouse *Tff3*. Intron No. 2 is followed by a third exon coding rCYSx12. The transgene is driven by the Tff3 promoter. A floxed *Neo* cassette (orange) under the control of a hybrid promoter LacUV5-SV40 was inserted to select recombinant clones. The white rectangles represent the vector backbone. After stable transfection, the native MTX cell line and recombinant MTX-rCYSx12 clones were amplified and cocultured at five ratios to obtain a range of rCYSx12 production. Production and secretion of rCYSx12 in a dose-dependent manner was observed by immunofluorescence of cell cocultures and by immunohistochemistry. (**c**) Distribution of beads in the mucus layer from MTX and MTX-rCYSx12 cells after 45 min sedimentation. (**d**) Beads distribution as a function of the mucus depth in MTX and MTX-rCYSx12 cultures. The width of each blob is proportional to the number of beads in each mucus section.

Mucus is a hydrogel comprising mainly water (>90%). Gel-forming mucins (GFMs) form a family of conserved proteins with a multi-domains structure. GFMs are the main mucus organic components and are responsible of physical properties of the mucus gels^8,9^. There are five human GFMs named MUC2, MUC5AC, MUC5B, MUC6, and MUC19, which form the mucus protein scaffold. These mucins are conserved in mice (Muc2, Muc5ac, Muc5b, Muc6 and Muc19). Among them, the association end-to-end of MUC2, MUC5AC and MUC5B *via* disulfide bonds to form long linear polymers is well characterized^10–12^. The large central moiety (>4000 amino acids) of each GFM monomer is rich in serine and threonine residues decorated with numerous *O*-glycans (Fig. 1a) ranging from 8 to 18 monosaccharides, which are responsible for mucus swelling upon secretion^13,14^.

So far, most studies have focused on well-characterized covalent interactions of GFMs. However, reversible interactions are likely crucial in the mucus ultrastructure, but difficult to study^15,16^. Along the GFM peptide axis, one of the best candidates to support transient interactions between GFM polymers is the “CYS” domain. This domain contains 10% of perfectly conserved cysteine residues and is highly conserved from invertebrates to humans^17–19^. For example, the pelagic tunicate *Oikopleura dioica* secretes Oikosin1, a molecule comprising 13 CYS domains to build a mucus “house”, which filters nutrients from seawater^20^. Human and mouse intestinal MUC2/Muc2 and human respiratory MUC5AC and MUC5B GFMs possess 2, 9, and 7 CYS domains, respectively. The amino-acid sequence of the CYS domain is composed of ~110 amino acids. Most of them are hydrophobic residues forming a globular structure maintained through intrachain disulfide bonds. The CYS domain intersects the central part of GFMs resulting in an alternation between hydrophilic highly *O*-glycosylated regions and hydrophobic CYS domains. This supports a possible role in mucus architecture by non-covalent self-associations and interactions with other molecules of the mucus content such as lipids^5,15,16,19,21–23^. More recently, we suggested that the CYS domain regulates mucin trafficking through the presence of *C-*mannose^24^.

The formation of hydrophobic intermolecular interactions between GFM polymers is strongly correlated with the gelation phenomenon^23^. It is believed that the CYS domain could be engaged in reversible hydrophobic interactions and thus participates in GFM interactome^25^. The aim of this study was to probe the potential of a molecule made of 12 consecutive identical copies of the CYS domain (rCYSx12) in stiffening mucus from HT29-MTX cells (MTX) secreting mainly the respiratory GFM MUC5AC (9 CYS domains), and mucus from scrapped mouse colonic mucosa for which Muc2 (two CYS domains) is the major, if not the only one, GFM. We found a reduction of permeability to bacteria-sized beads and a decrease of bacterial and mouse spermatozoa motility (swimming speed and linearity) in a dose-dependent manner, but independently of the microbiota, and a reduction of the colonic mucus mesh size associated with an increase of local viscosity probed by particle diffusion. Our present data demonstrate that the delivery of rCYSx12 leads to the stiffening of the GFMs network, which makes the mucus more protective toward potentially deleterious environmental components.

## Materials and Methods

### Recombinant MTX cells

A mucin-secreting MTX cell subpopulation was stably transfected with an 18.1-kb expression plasmid vector previously used to generate a transgenic (Tg) line^26^ and slightly modified to insert a neomycin phosphotransferase cassette to select positive MTX cells. Details concerning the vector construct, cell culture, and recombinant cell line are available in the Online Resource 1. Recombinant cells were used before the 20^th^ passage as we observed a decrease of the rCYSx12 production after 20 passages (data not shown).

### Characterization of MTX:MTX-rCYSx12 cocultures

MTX cells were routinely cocultured until confluence with MTX-rCYSx12 clone at five different ratios (%MTX:%MTX-rCYSx12): 100:0; 75:25; 50:50; 25:75; and 0:100, before assessing production and secretion of the recombinant molecule, so that bacteria motility could be studied. Recombinant CYSx12 production was checked in the MTX:MTX-rCYSx12 cocultures using immunofluorescence and Western blotting as described in the Online Resource 1 section with an antibody that recognizes a short peptide of the CYS domains Nos. 2, 3, and 5 of MUC5B^27^. The procedures of rate zonal centrifugation, determination of MUC5AC expression by quantitative RT-PCR and production by dot blot, and immunohistochemistry were described in the Online Resource 1.

### Colonic mucus

The mouse Tg line secreting rCYSx12 in its gut lumen^26^ was maintained by breeding heterozygous Tg mice with C57BL/6 wild-type (WT) mice. Tg and their control WT littermates were kept in the specific pathogen-free animal facility of the University of Lille. Housing conditions were compliant with the European guidelines for animal welfare. All procedures were in accordance with the French Guide for the Care and Use of Laboratory Animals and with the guidelines of the European Union. The Tg line has been registered under the GMO number 5287 at the French Minister of Education and Research. Mice were genotyped as previously described^26^ and used when they were aged from 9 to 12 weeks. To mitigate environmental effects, pairs of mice were used, i.e., brother–brother or sister–sister siblings from the two genotypes, and housed in the same cage. Mice were fasted 12 h before experimental procedures. They were killed by cervical dislocation. Genotypes were systematically observed with the green fluorescent protein tag of the transgene in fresh intestinal tissue, using an epifluorescence microscope (M205, Leica) equipped with a color camera (DFC450c, Leica). Muc2 concentration was determined by densitometry as described in Online Resource 1. In order to determine the gel properties, colonic mucus was recovered by gentle scraping of the whole colonic mucosa after removing feces. Two mucus groups were prepared from three to five mice per genotype. Mucus samples were diluted 1:1 (w/w) with phosphate buffered saline (PBS, Gibco BRL) containing AEBSF (1 mM, Sigma-Aldrich) protease inhibitor and mucus samples were characterized immediately after sampling.

### Mucus permeability

Three micrometer diameter yellow-green (YG) carboxylated beads (Polysciences) were used to measure mucus permeability. The cells were cultured in four chamber slides for at least 3 weeks (Labtek). The medium volume was reduced to 200 µL and 3 µL of the unmodified bead solution (dilution 1/100) was loaded on the cell surface of MTX and MTX-rCYSx12 cells. The cell layer was stained with CellTrace Bodipy TR Methyl Ester (dilution 1/1000, Invitrogen). Bead distribution within the mucus layer was determined after 45 min of sedimentation (37 °C, 5% CO_2_) with an inverted confocal microscope (Spinning disk, magnification x20, Zeiss) at a resolution of 4 µm under the *z*-axis from the cell surface to the last luminal mucus section defined as the last section with beads at the time of loading. The determination of bead number in each mucus section was performed with Fiji. We considered that the beads that reached the first third of all *z* sections nearest the cell surface have effectively crossed the mucus layer.

### Particle tracking microrheology

Two hundred nm and 1 µm diameter YG beads (dilution 1/100) bound to low-molecular-weight diamine-poly(ethylene glycol) (PEG) (3.4 kDa, Sigma-Aldrich) were used according to the manufacturer’s protocol and as described elsewhere^28,29^. The efficient linkage between the carboxyl group of the beads and the free amine on the PEG was confirmed by incubation with fluorescent avidin, which cannot be adsorbed on beads if they are coated with PEG. The size (expressed as Z-average) and the charge (i.e., the ζ-potential) harbored by the beads in PBS were assessed in triplicate at 25 °C using dynamic light scattering (ZetaSizer NanoZS analyzer, Malvern Instruments). Two µL of the bead solution were loaded in about 20 µL of fresh mucus. Samples were placed at 37 °C to record the bead motions at a temporal resolution of 20 frames/s (fps) on 10 different fields with an inverted microscope (Spinning disk, magnification x100, Zeiss) equipped with a CCD camera (ImagEMX2, Hamamatsu). Particle tracking was then analyzed using the Trackmate plug-in from Fiji software and the bead coordinates were transformed into time-averaged mean square displacement (MSD) with Icy software^30^ using the formula:

$$MSD={[(x+\varDelta )-x]}^{2}+{[(y+\varDelta )-y]}^{2}$$

where *x* and *y* are the coordinates of each particle centroid, and Δ the time lag. MSD curves were represented as average ± sem (standard error of the mean). We calculated the mean diffusivity *D*_eff_ from the MSD at τ = 1 s using the following formula suitable for 2-D tracking^31^:

$${D}_{eff}=\frac{MSD}{{\rm{4}}\tau }$$

where τ is the time scale. The same procedure was used for 1 µm PEG-coated beads except concerning the recording temporal resolution which was 50 fps. The obstruction-scaling model was applied to estimate the polymer mesh sizes^32^. This model relies on the diffusivity of a solute, which does not interact with polymers, to the obstruction encountered (i.e. the impediment coming from the polymer network to Brownian diffusion). This model was applied to characterize mucus mesh size using the equation^32,33^:

$$\frac{{D}_{g}}{{D}_{{\rm{0}}}}=\exp [-\frac{\Pi }{{\rm{4}}}{(\frac{{r}_{s}+{r}_{f}}{{r}_{g}+{r}_{f}})}^{{\rm{2}}}]$$

where *r*_*s*_ is the bead radius, *r*_*f*_ is the estimated radius of mucin fibers, *D*_*g*_ the measured diffusion coefficient in the gel, and *D*_0_ the theoretical diffusion coefficient in water. We assumed that *r*_*f*_ is 3.5 nm^5,34^. Therefore, the mucus pore size was 2 × *r*_*g*_. We empirically considered 200 nm diameter particles as strongly hindered if their *D*_eff_ < 0.032 µm^2^/s at τ = 0.25 s, which corresponds to a displacement of less than the diameter of the bead^31^.

### Bacterial motility

A fluorescent PhoPc strain of *Salmonella enterica* serovar Typhimurium (gift from Dr. J.-C. Sirard, Institut Pasteur de Lille, France) and fluorescent PAO1 strain of *Pseudomonas aeruginosa* (gift from Dr. B. Borlee, Colorado State University, USA) were grown separately in 5 mL of Luria–Bertani broth medium containing ampicillin (50 µg/mL, Sigma-Aldrich) for 12 h before each experiment^35,36^. Bacterial suspension was diluted in PBS to 10^8^ bacteria/mL using OD_600_. To determine bacterial motility in mucus from MTX cell cocultures, the volume of DMEM lining the cell layer was reduced to 100–200 µL and 6 µL of *P. aeruginosa* suspension was then added. Two µL of *S. enterica* suspension was added to about 100 µL of mucus scraped from the whole colon of mice. Motility was recorded using an inverted microscope (MM AF 1.3, magnification x40, Leica) at a resolution of 4 fps for 60 s. Tracks with a duration shorter than three frames (0.5 s) were excluded from analysis. Six records per condition (order of recording was alternated) were analyzed using a Trackmate plug-in from Fiji for tracking and Icy software for trajectories analysis. Swimming speed and linearity were quantified to determine quantitative and qualitative features of cell displacement, respectively. The swimming speed was calculated as the ratio of total displacement over the displacement duration whereas the linearity is the distance of the track (linear distance from the first to the last point of the trajectory) over its length (total trajectory distance). Thus, the displacement can be categorized as linear (linearity >0.7), curvilinear (0.3< linearity <0.7) or confined (linearity <0.3). More the linearity decreases below 0.3 more the displacement in mucus is confined reflecting a steric hindrance and/or interactions with the matrix^37^.

### Spermatozoa motility

Mouse spermatozoa were recovered from minced cauda epididymis of two WT mice. The tissue was incubated in 1 mL of prewarmed M2 medium (Sigma-Aldrich) at 37 °C for 10 min allowing spermatozoa to swim free of the minced tissue. To isolate mature spermatozoa, the M2 medium supernatant was placed on a discontinuous 45–90% Percoll gradient buffered with TL-HEPES (pH 7.4) prepared as described elsewhere^38^ and centrifuged at 300 *g* for 30 min. The spermatozoa pellet was incubated (10 min, 37 °C, 5% CO_2_) in 200 µL of prewarmed M2 containing 0.01% acriflavine (Sigma-Aldrich). For each condition, 10 videos were recorded on an inverted microscope (Spinning disk, magnification x20, Zeiss) at 37 °C, 5% CO_2_. The analysis method was identical to that for bacteria tracking.

### Statistics

MSD curves were represented as means ± sem. Comparisons of bacteria, sperm cells motility and mucus mesh sizes were carried out using Wilcoxon–Mann–Whitney test. Comparisons of bead diffusion were performed with one tailed *t*-test. All statistics were carried out with R software. *P* < 0.05 was considered significant.

## Results

### Characterization of MTX:MTX-rCYSx12 cocultures

We reported previously that the intestinal mucus of the Tg mouse that secretes rCYSx12 is less permeable to inert particles^39^. However, the underlying mechanism is difficult to understand because of the interdependence between the microbiota composition and the *O*-glycosylation of MUC2^14,26^. To circumvent the putative microbiota effect, we stably transfected the mucus-secreting MTX cell line with an expressing vector to secrete rCYSx12 (MTX-rCYSx12) and characterized the cocultures by immunofluorescence, Western blot and rheological techniques (Fig. 1b). Recombinant CYSx12 secretion in sterile MTX:MTX-rCYSx12 cocultures at five different ratios (%MTX:MTX-rCYSx12, 100:0; 75:25; 50:50; 25:75; and 0:100) was first assessed by immunofluorescence. Quantification of CYS-positive cells showed that rCYSx12 was increased in the five cocultures in a dose-dependent manner (Online Resource 2a,b). We next confirmed by Western blotting that rCYSx12 is produced in cell lysate of cocultures with MTX-rCYSx12 cells and secreted in a dose-dependent manner at a molecular mass of about 170 kDa (Online Resource 2c,d), as consistent with its calculated molecular mass of 167 kDa. We next examined whether rCYSx12 was intimately associated with MUC5AC (9 CYS domains), which is the main GFM of MTX cells^40^. Fractions collected after rate zonal centrifugation of the mucus from MTX and MTX-rCYSx12 cultures were analyzed using MUC5AC-specific antibody and revealed a typical sedimentation profile for the mucus of control MTX cells with a single peak (Online Resource 2e). A modified MUC5AC density distribution for mucus from MTX-rCYSx12 with two mucus populations was evidenced with one containing both rCYSx12 and MUC5AC showing a close association between MUC5AC and rCYSx12 (Online Resource 2e). The production of rCYSx12 was independent of MUC5AC genetic expression and secretion as revealed by quantitative RT-PCR, immunoblot and immunohistochemistry (Online Resource 3a–c). These data demonstrated that MUC5AC concentration was similar in the 5 coculture conditions.

### Recombinant CYSx12 secretion induces a decrease of the gel permeability

Because the mucus architecture is crucial for protecting the underlying epithelium from exogenous particles, we first examined the permeability of mucus using fluorescent micro-beads. We determined the sedimentation profile of inert beads loaded at the surface of MTX and MTX-rCYSx12 cultures. The distribution of 3 µm beads within the mucus layer after 45 min of sedimentation highlighted an increase in the density of the beads in the upper mucus sections with rCYSx12 production (Fig. 1c,d). The percentage of beads that crossed the whole mucus layer of MTX and MTX-rCYSx12 cultures decreased from 81.5% to 72.1%, respectively. Since, rCYSx12 has an effect on mucus structure and permeability we hypothesized that active displacements of mucus-associated bacteria may be hindered by rCYSx12 enrichment.

### Recombinant CYSx12 enrichment decreases bacterial motility *in vitro*

**Figure 2:**
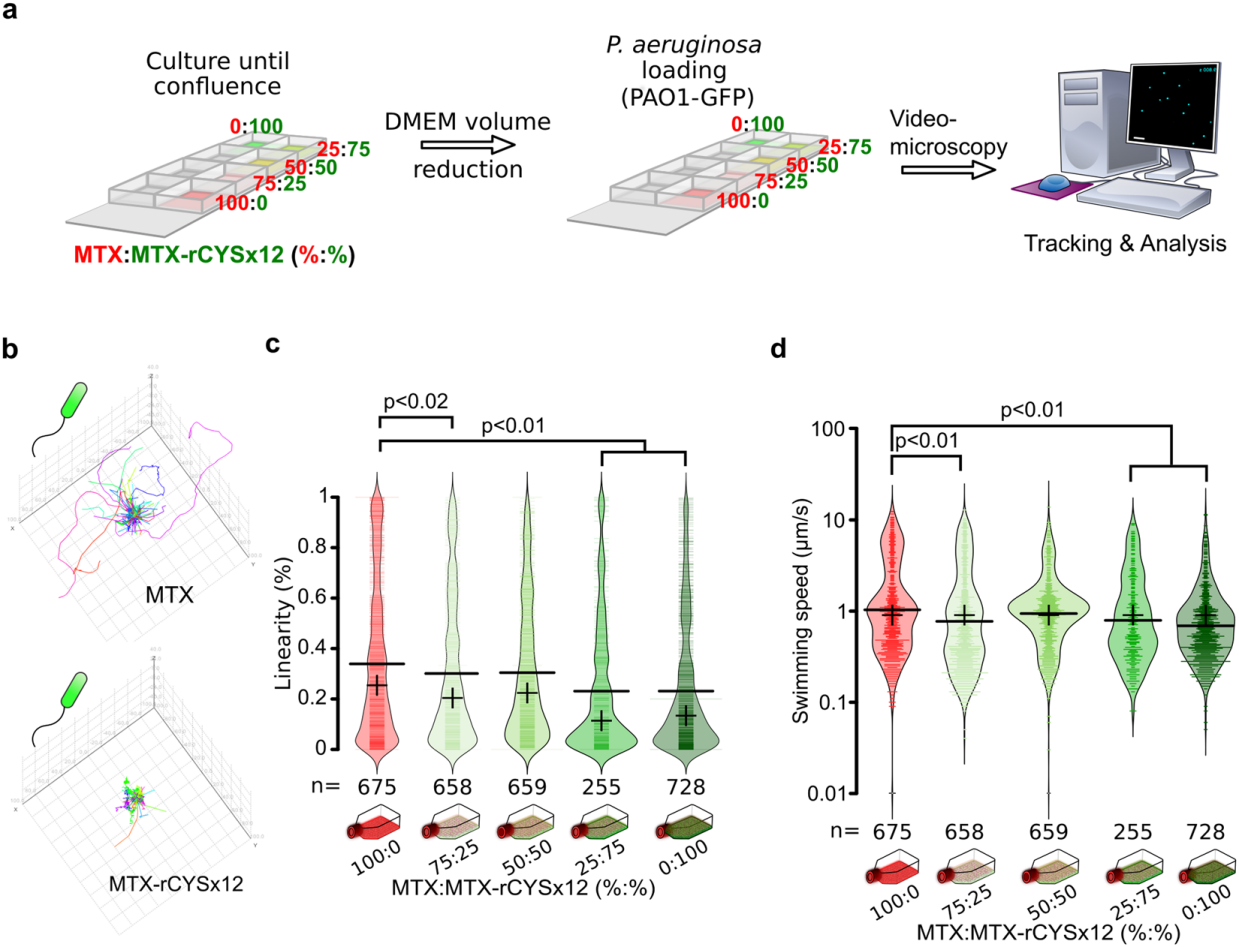
Decreased motility of *P. aeruginosa* in MTX mucus coculture enriched in rCYSx12. (**a**) Study design for the analysis of motile cell displacements in mucus overlaying MTX:MTX-rCYSx12 cocultures. Tracking and analysis were performed with Fiji and Icy, respectively. (**b**) Representative trajectories of *P. aeruginosa* in the mucus overlaying MTX and MTX-rCYSx12 cells. (**c**) Analysis of *P. aeruginosa* linearity in the mucus of the 5 MTX:MTX-rCYSx12 cocultures *in situ* using bean plots. (**d**) Analysis of *P. aeruginosa* swimming speed in the mucus of the 5 MTX:MTX-rCYSx12 cocultures *in situ* using bean plots. For (**c**,**d**), average and median are indicated by a horizontal bold line and a cross, respectively. The number (*n*) of tracked bacteria is given at the bottom of the figure and the statistical analyses were conducted using a two-sided Wilcoxon-Mann-Whitney test. Results are representative of three independent experiments.

To assess the ability of rCYSx12 to limit unicellular motility in mucus, displacement of a fluorescent bacterial strain of *Pseudomonas aeruginosa*, a bacteria frequently found in airways mucus from cystic fibrosis patients, was studied by videomicroscopy in the mucus of the five cell coculture ratios (Fig. 2a). Figure 2b and Online Resource 4 illustrate the clear difference in bacterial swimming behavior between the MTX and MTX-rCYSx12 cultures from one set of experiments highlighting more confined bacterial trajectories in rCYSx12-enriched mucus. The calculated linearity (ratio of distance over length) of trajectories decreased significantly in the mucus enriched in rCYSx12, as outlined in Fig. 2c. The average linearity decreased (–32%) from 0.34 for MTX mucus to 0.23 for MTX-rCYSx12. The swimming speed of bacterial displacement also decreased significantly (47%) from 1.92 to 1.01 µm/s as a result of rCYSx12 enrichment (Fig. 2d).

### Recombinant CYSx12 enrichment leads to a decrease of bacteria motility *ex-vivo*

**Figure 3:**
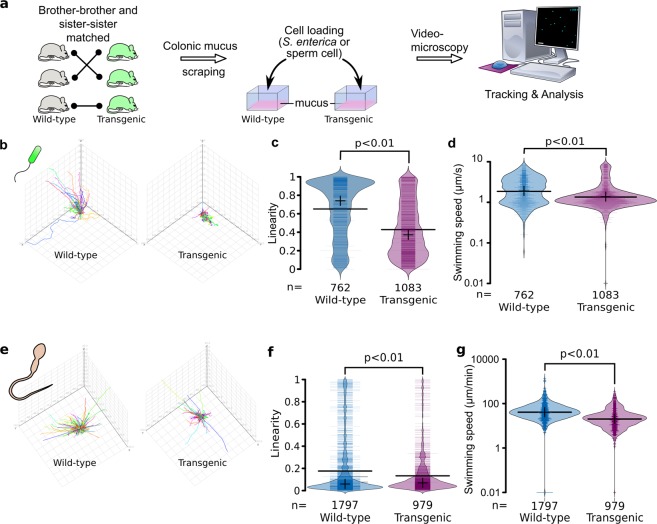
Decreased cell motility in rCYSx12-enriched mucus scraped from mouse colons. (**a**) Study design for the analysis of motile cell displacements in mucus scraped from the colons of mice. Tracking and analysis were conducted with Fiji and Icy, respectively. (**b**) Representative trajectories of fluorescent *S. enterica* in the mucus from wild-type and transgenic mice. (**c**) Analysis of *S. enterica* linearity in mucus from wild-type and transgenic mice *ex vivo* using bean plots. (**d**) Analysis of *S. enterica* swimming speed in mucus from wild-type and transgenic mice *ex vivo* using bean plots. (**e**) Representative trajectories of spermatozoa in the mucus from wild-type and transgenic mice. (**f**) Analysis of spermatozoa linearity in mucus from wild-type and transgenic mice using bean plots. (**g**) Analysis of spermatozoa swimming speed in mucus from wild-type and transgenic mice using bean plots. For (**c**,**d**,**f**,**g**), and g, average and median are indicated by a horizontal bold line and a cross, respectively. The number of tracked cells (*n*) is indicated at the bottom of the figure and the statistics were conducted using a two-sided Wilcoxon-Mann-Whitney test. Results are representative of three independent experiments.

We next examined if the colonic mucus from Tg mice secreting in the intestine rCYSx12, with the GFM Muc2 (two CYS domains), hinders also motile cell displacement in comparison to WT littermates. We previously reported that expression level of the major GFM *Muc2* measured by quantitative RT-PCR is similar between the two genotypes. Here we checked Muc2 production by semi-quantitative dot blot densitometry and found Muc2 to be produced at similar levels between mucus samples from WT and Tg mice (Online Resource 3d). To study bacterial displacements in mucus scraped from the whole colon of mice, we first used a fluorescent *Pseudomonas aeruginosa* strain and found that the strain was not able to swim in the mouse colonic mucus (data not shown). Therefore, we decided to study the motility of a fluorescent *Salmonella enterica* bacterial strain, which is more relevant for the intestinal tract. Colonic mucus samples from control WT and Tg mice were examined using videomicroscopy (Fig. 3a). Figure 3b and Online Resource 5 illustrate bacterial trajectories in the two mucus samples highlighting more confined trajectories in the mucus enriched in rCYSx12. These data are in agreement with our observations using mucus from MTX cocultures. Indeed, displacement of *S. enterica* calculated from hundreds of trajectories demonstrated that rCYSx12 secretion is associated with a significant 34% decrease of swimming linearity (Fig. 3c). This more confined bacterial displacement was also associated with a significant 29% decrease of the bacteria swimming speed from 2.41 µm/s in the colonic mucus of WT mice to 1.70 µm/s in the mucus from Tg mice (Fig. 3d).

### Recombinant CYSx12 enrichment leads to a decrease of spermatozoa motility *ex-vivo*

In female genital tract, spermatozoa motility is highly dependent on mucus structure^5^. To assess their displacements in a mucus gel enriched with poly-CYS molecules, mature spermatozoa isolated from WT mice were first incubated with acriflavine to render them fluorescent for videomicroscopy tracking. No evidence for change of spermatozoa staining and/or viability was observed between the beginning and the end of the experiment (data not shown). As for *S. enterica*, spermatozoa trajectories were significantly more confined in rCYSx12-enriched colonic mucus than in mucus from WT mice, as illustrated in Fig. 3e and Online Resource 6. The calculated linearity decreased significantly from 0.54 to 0.38 (−30%) for the mucus of WT and Tg mice, respectively (Fig. 3f). The more confined trajectories of the spermatozoa in the mucus of Tg mice were also associated with a significant 67% decrease of mean swimming speed from 139.5 to 46.9 µm/min (Fig. 3g). Together, these results provided clear evidence that the delivery of rCYSx12 into the mouse mucus hindered motile cell displacement. To better understand how poly-CYS molecules hinder motile cell displacements we next used particle tracking microrheology.

### Recombinant CYSx12 enrichment leads to mucus mesh size tightening

**Figure 4:**
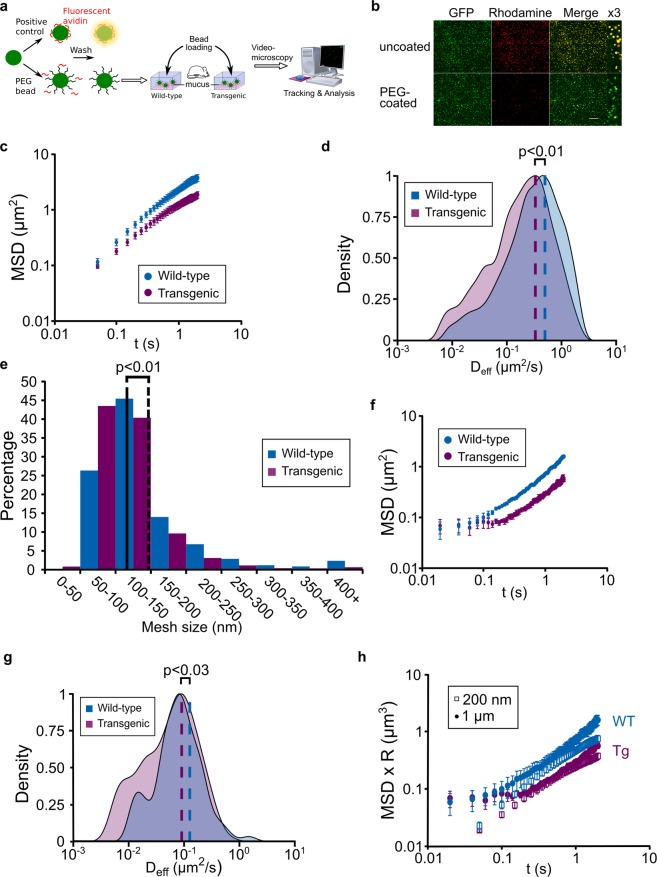
Microrheological analysis of CYS domain-enriched mucus scraped from mouse colons. (**a**) Study design for the analysis of PEG-bead diffusion. After checking their PEG coating, fluorescent beads were loaded in mucus scraped from the colons of wild-type and transgenic mice. Tracking and analysis of bead diffusion were conducted using Fiji and Icy, respectively. (**b**) Coating of 200 nm diameter YG beads (green fluorescence) with low-molecular-weight PEG. Effective coating was checked by incubation with avidin–rhodamine (red fluorescence). The avidin adsorbs onto uncoated beads (yellow fluorescence on merged pictures), but not beads coated with PEG (see the x3 enlargements). Scale bar = 50 µm. (**c**) MSD ± sem as a function of time for 200 nm PEG-coated beads embedded in mucus from wild-type (in blue) or transgenic (in purple) mice. (**d**) Diffusion coefficients distribution (t = 1 s) of 200 nm fluorescent beads embedded in colonic mucus from wild-type (in blue) or transgenic (in purple) mice. The average diffusion coefficient is represented by the vertical dashed line. (**c**,**d**) Data are averaged from 5 independent experiments. (**e**) Mucus mesh-size distribution calculated with the obstruction-scaling model in mucus from wild-type and transgenic mice. Data were pooled from 5 independent experiments. (**f**) MSD ± sem as a function of time for 1 µm PEG-coated beads embedded in mucus from wild-type (in blue) or transgenic (in purple) mice. (**g**) Diffusion coefficients distribution (t = 1 s) of 1 µm fluorescent beads embedded in colonic mucus from wild-type (in blue) or transgenic (in purple) mice. The average diffusion coefficient is represented by the vertical dashed line. (**f**,**g**) Data are averaged from 3 independent experiments. (**h**) MSD of 200 nm (filled circle) and 1 µm (empty square) particles in mucus of wild-type (WT, blue) and transgenic (Tg, purple) mice scaled by their radius R. (**d**,**g**) Diffusion coefficients lower than the uncertainty were excluded from the analysis.

Assessing modifications of mucus structure is challenging. To gain further insight into the modification of GFM architecture by rCYSx12 enrichment, we decided to estimate the pore size of the GFM network using beads. YG beads (200 nm diameter) covalently bound to low molecular mass PEG were used to probe the particle diffusion at the nanoscale and microscale levels^33^. The design of the experiment is depicted in Fig. 4a. The PEG confers a near-neutral bead surface strongly reducing their interactions with GFM and rendering their diffusion in the gel. Appropriate PEG coating was confirmed by incubating the coated beads with rhodamine–avidin (Fig. 4b) and by assessing ζ-potential (Online Resource 7). The PEG coating acted as a shield against the negative ζ-potential as observed by Lai *et al*.^28^. PEG-coated beads were loaded into freshly harvested colonic mucus and their diffusion was tracked by videomicroscopy 10 min later. Beads displayed a higher diffusion rate in the colonic mucus from WT mice than in mucus from Tg mice as outlined by the MSD curves averaged from five independent experiments (Fig. 4c, Online Resource 8). The calculated mean diffusion coefficient was 0.580 µm^2^*/*s for mucus from WT mice, which is 3.8-fold slower than their theoretical diffusion coefficient in water, and 0.310 µm^2^*/*s for the rCYSx12-enriched mucus, which is 7.1-fold slower than in water. A higher number of beads displaying low diffusion coefficient was observed in mucus from Tg mice compared to WT littermates showing that mucus enriched in rCYSx12 is more obstructive to diffusion (Fig. 4d). In mucus from WT and Tg mice, 4.7% and 13.4% of particles were hindered (D_eff_ < 0.032 µm^2^*/*s at t = 0. 25 s), respectively. We next applied the obstruction-scaling model to the diffusion coefficients measured from five independent experiments. Distribution in mesh size of colonic mucus from WT and Tg mice is given in Fig. 4e. Consistent with the literature^41^, more than 95% of mucus pores were <300 nm. However, there was a significant 20.3% decrease of the average mesh size in mucus from Tg compared with WT mice with a mean of 148 and 118 nm for WT and Tg mice, respectively. The analysis of mesh size profile revealed a clear increase of 0–100 nm meshes subpopulation in the mucus of Tg mice compared to WT littermates (Fig. 4e).

In an attempt to approximate the physical environment encountered by micrometric objects such as bacteria in mucus, 1 μm particles were next used. In agreement with MSD curves obtained with 200 nm particles, a lower diffusion rate was found in mucus enriched in rCYSx12 highlighting a more obstructive mucus (Fig. 4f, Online Resource 9). The averaged diffusion coefficient decreased from 0.181 to 0.073 µm^2^*/*s (−59.7%) in mucus from WT and Tg mouse, respectively (Fig. 4g). The product of the bead radius (R) and MSD plotted over time were very close to each other indicating the absence of probe size effect on diffusion measurement (Fig. 4h)^42^. However, the bead diffusion rate in mucus depended on their size in agreement with previous reports from others^43,44^. It is noteworthy that similar results were obtained by applying the obstruction scaling model at a length scale of 1 μm particle. The average mucus mesh size were 140 and 122 nm for mucus from WT and Tg mouse, respectively. Together, these data demonstrated a stiffening of the GFM network by rCYSx12, which hinders particles and motile cells in the gel.

## Discussion

During the past two decades, remarkable progress has been made in research on mucus gels and showed that GFMs form entangled networks by disulfide bridges between GFM monomers^10–12^. However, little work has been done to assess the nature of nondisulfide bond links between mucin polymers because these interactions are versatile. The hydrophobic nature of the CYS domain together with its presence several copies in GFMs of all secretory mucosa^6^ suggest a role in reversible associations of GFMs. However, such interactions remain difficult to study despite their potential involvement in mucus dynamics^16,45^. To date, the association of CYS domains in noncovalent dimers and transglutaminase 2 dependent covalent association was investigated using recombinant molecules obtained from cell culture^25,46^. However, accurate analysis of interactions between GFMs requires the maintenance of their structure *in vivo*. Although our strategy used endogenous delivery of poly-CYS at an unknown concentration to investigate, it underlines the possibility to modify the biophysical and physiological properties of mucus *in vitro* and *ex-vivo*. The mucus from MTX cell cocultures and from mice allow to get insight in the properties of mucus for which the mucin network is made mainly of MUC5AC with nine CYS domains and Muc2 with two CYS domains, respectively.

Previously, we reported that mucus enrichment with rCYSx12 strengthened the colonic mucus from Tg mice. However, it was difficult to prove a direct effect of rCYSx12 on the alteration of mucus properties as it was accompanied by a change of gut microbiota composition, which also play a direct role in the mucus properties^25^. To bypass these effects, we developed a biological model using sterile cocultures with mucus-secreting MTX cells and recombinant MTX secreting rCYSx12. Because the diffusional transport is known to be negligible over the sedimentation for particles >1 µm due to their increase of inertia^47^, we used 3 µm-diameter beads to perform sedimentation experiments. Importantly, using mucus from cell culture, both particle sedimentation and motile cell displacement decreased with rCYSx12-enrichment in a dose dependent manner for cell motility.

The cellular model showed that density distribution of the main GFM MUC5AC in MTX, differs with rCYSx12 enrichment as we found a shift of MUC5AC to bottom fractions of the sucrose gradient for MTX-rCYSx12 mucus. It indicates the formation of denser structures. A similar phenomenon has been reported in salivary mucus from healthy humans who consumed green tea polyphenol dietary supplements^48^. Thse tendency to the CYS domain to self-aggregate together with and increase of its concentration in rCYSx12 enriched mucus is likely responsible for a remodeling of the GFM network resulting in a change of the mucus structure^49^. This suggests that some of GFM CYS domains are available for interactions in the mucus matrix.

The particle diffusion probed at the nano- and microscale levels allows to approach the local environment encountered by molecules and bacteria^50^. MSD of beads is linked to mesh size of the GFM network, but other macromolecules, such as DNA and F-actin for example, may also contribute to the changes observed [6]. In the present study, mucus samples were diluted 1/1 (w/w) with PBS to normalize its pH. As expected, this led to an increase in transport rates compared with other studies that used undiluted mucus^6,15,41,45,51^. The aging effect of the mucus samples on the rheological properties is an important factor, but this was weakened by alternating systematicaly the order of samples between each replicate. These experimental conditions allowed us to evidence an effect of mucus rCYSx12 enrichment.

Particle diffusion that depends on the mucus ultrastructure^15,43^ was studied using mucoinert particles that diffuse through mucus gels more easily than uncoated beads^28^. We cannot rule out that the persistence of residual polar groups at the particle surface may occur. However, the magnitude of the interactions between these groups and GFMs was identical between mucus from WT and Tg mouse. Mucus are dynamic gels maintained by physical interactions, which are influenced by the physicochemical environment^6,52^. Thus, mucus gels are heterogeneous materials composed by transient relaxed and unrelaxed clusters, which break and reform across time allowing particles larger than GFM mesh size of unrelaxed clusters to diffuse in the gel for long timescales^15,42^.

The subdiffusive regime in rCYSx12-enriched mucus, indicated by the MSD measured using 0.2 and 1 µm diameter particles, showed a mucus stiffening with a stronger resistance to particle diffusion than mucus without such enrichment. This change was confirmed using the obstruction-scaling model. The mucus mesh sizes of WT mice were found similar to those reported by others from 100 to 300 nm^39,44^. In rCYSx12 colonic mucus, we observed an increase number of 50–100 nm pores (Fig. 4e) responsible for the decreased bead diffusion and hindrance of swimming cell displacement.

Most swimming pathogens have developed complex machines to propel them across the mucus layer with the aim to reach, colonize, or invade epithelial tissue. To prevent the bacterial burden, GFMs form a sticky glycoprotein network to entrap them. We found that bacterial motility was dependent on the mucus sample. *P. aeruginosa* was motile in mucus from MTX, for which MUC5AC is the major GFM, but not in colonic mucus. Despite their various sizes (3 µm width for spermatozoa and 1 µm for *S. enterica*), we found a greater number of bacteria and spermatozoa trapped in CYSx12-enriched mucus as highlighted by the decrease of swimming linearity and speed. While the bacteria/sperm cells speed reflects the energy needed to clear a path through mucin polymers, the linearity is a good indicator of the heterogeneity of the gel. However, an increase of viscoelasticity alone is probably not sufficient to explain this phenomenon because, at physiological viscoelasticity, bacteria and spermatozoa are able to swim^53–55^. Nevertheless, a drastic increase of viscoelasticity, which is a hallmark of the airway mucus in individuals with cystic fibrosis, reduces both particle diffusion and bacterial motility^56^. Thereby, the formation of smaller pores measured by microrheology likely plays a synergistic role together with the alteration of mucus microrheology in the hindrance of motile cells. This natural phenomenon was demonstrated in the endocervix during the periovulatory period of the menstrual cycle preventing spermatozoa passage^5^. Similarly to what has been observed for the delivery of lipids in mucus, direct interactions between the transgene rCYSx12 and motile cells can not be ruled out^57^.

In conclusion, rCYSx12 is critical for the biophysical behavior of the mucus hydrogel supporting a fundamental function of the CYS domain in the mucus properties. Here, we demonstrate that the delivery of a molecule made only of CYS domains stiffens the GFM network, which in turn makes the gel more obstructive to bacteria and sperm cells. The correlation between mucus ultrastructure and beads diffusion is well known^58^ and our findings highlight the possibility of altering the mucus barrier through the delivery of a mucin-based hydrophobic molecule. However, the exact mechanism involved in mucus strengthening needs further research. Our hypothesis was that the CYS domain delivery increases interactions between mucins and/or components of the mucus matrix through low energy forces leading to a stiffening of the mucin network with denser meshes. Thereby, recombinant CYS domain delivery could help to determine the minimal amount of recombinant protein necessary to reinforce the colonic mucus with aim to limit diffusion of macromolecules and toxins in mucus as observed for the delivery of lipids^59^. Reinforcing mucus hydrogels is an attractive approach which can be achieved through the delivery of recombinant molecules^60^ or by administering recombinant probiotics that would secrete molecules made of CYS domains. This strategy could help in reducing the bacterial burden in the context of mucus-associated diseases, such as ulcerative colitis^61^. Conversely, a better understanding of the CYS::CYS or CYS::mucin interactions could help to develop new mucolytics to inhibit such interactions^39^.

## Supplementary information

Online Resources 1-3, 7 and 10

P. aeruginosa trajectories in mucus from MTX (left) and MTX-rCYSx12 (right)

S. enterica trajectories in scrapped mucus from WT (left) and Tg (right) mice

Sperm cell trajectories in scrapped mucus from WT (left) and Tg (right) mice

Online Resource 7

200 nm diameter bead diffusion in mucus from WT (left) and Tg (right) mice

1 µm diameter bead diffusion in mucus from WT (left) and Tg (right) mice
