# Supplementary material for: Mucin CYS domain stiffens the mucus gel hindering bacteria and spermatozoa
Source: Sci Rep. 2019 Nov 18;9:16993. doi: 10.1038/s41598-019-53547-x (PMC6861317; doi:10.1038/s41598-019-53547-x)
Supplement: Supplementary file 1 — Online Resources 1-3, 7 and 10 [file 41598_2019_53547_MOESM1_ESM.pdf]

Mucin CYS domain stiffens the mucus gel hindering bacteria and spermatozoa

Short title: Mucin CYS domain stiffens the mucin network

Bastien Demouveaux, Valérie Gouyer, Catherine Robbe-Masselot, Frédéric Gottrand, Tetsuharu Narita and Jean-Luc Desseyn

Online Resource 1. Supplemental Material & Methods.

Generation of recombinant HT29-MTX cell line

Two novel cell lines derived from HT29 cell clones were generated to express stably a recombinant molecule made of 12 copies of a CYS domain (rCYS×12) from the human MUC5B gel-forming mucin. The mucus-secreting cell line, HT29-MTX-E12 (MTX) was purchased (ECACC, UK). Cells were grown in DMEM high glucose media (Dulbecco's Modified Eagle's Medium) supplemented with 862 mg/mL GlutaMAX (Gibco BRL), 10% (v/v) heat-inactivated fetal calf serum (Thermo Scientific, France), and a mix of 100 U/mL of penicillin and 100 μg/mL of streptomycin (Gibco BRL) at 37°C and 5% CO_2. Ten cm dishes were used for transfection experiments and uncoated T25 and T75 cm^2 flasks were further used (TPP, Techno Plastic Products). Media were changed every 48 h and experiments were performed using 100% confluent cultures at least 21 days after plating. A 2.3-kb *Xho*I/*Xho*I fragment containing a neomycin phosphotransferase (Neo) expression cassette under a hybrid LacUV5-SV40 promoter and flanked by two loxP sites was prepared for positive selection in MTX cells. The 18.1-kb No. 208 plasmid vector, which was used to create a transgenic mouse that secretes into its gut a recombinant molecule made of 12 consecutive CYS domains under the mouse Tff3 promoter was modified as follows. A 322-bp fragment was excised by digesting the plasmid with *Hpa*I and *Stu*I and religated. The plasmid was then linearized using *Sal*I. The final No. 208-Neo 20.2-kb expression vector was then obtained by subcloning the *Xho*I/*Xho*I Neo cassette into the unique *Sal*I site. Only the plasmid with the Neo transcription in the sense opposite to the rCYS×12 gene was used. Cells were transfected by 60 μL Effectene and 16 μL of amplified DNA solution with *Sac*II-linearized vector according to Manufecturer instructions (Qiagen). Forty eight hours after transfection,

medium containing G418 (Sigma-Aldrich) at 500 μg/mL, was added. Seven MTX G418-colonies were isolated and amplified, and assayed for the presence of the transgene by PCR using the two oligonucleotides 5´-ACCTACTCCAACATCCGTGC-3´ (forward) and 5´-GTAGGTGTCAAAGTCCCCGC-3´ (reverse). Positive cells were then screened for the expression of the transgene by RT-PCR using the two oligonucleotides 5´-GCTGCCATGGAGACCAGA-3´ (forward, within exon No. 1) and 5´-TGTGGATCCCCTCCAGCTG-3´ (reverse, within exon No. 3). The production of rCYS12 was then assessed by western blotting on three MTX clones. One MTX for which the production seemed higher was used for further experiments. The number of cells seeded was different for MTX and MTX-rCYS×12 to compensate for the difference in growth rate (440 000 cells/T25 for MTX and 1 200 000 cells/T25 for MTX-rCYS×12).

MUC5AC expression in MTX:MTX-rCYS×12 cocultures

Total RNA extraction, cDNA synthesis and PCR runs using 18S ribosomal RNA as an internal positive control were conducted in triplicate. The MUC5AC expression was determined in three different confluent MTX and MTX-rCYSx12 cultures by quantitative real-time RT-PCR. Primers and TaqMan probe sequences were selected using the Primer3 freeware. The oligonucleotides used were 5´-AGAGTGGGAGCTGGGAGAGAG-3´ (forward) 5´-AGCTCAGAGGACATATGGGAGGT-3´ (reverse) and probe was 5′-CCAGTGTCCCCCATGCAGTGACC-3′. The cycle threshold values were measured by the ABI Prism 7700 sequence detector system (Applied Biosystems) and MUC5AC expression levels were normalized to mRNA levels of 18S ribosomal RNA (TaqMan Ribosomal RNA Control Reagents, Applied Biosystems, USA). MUC5AC RNA amounts were determined using the ΔΔCt method.

Recombinant CYS×12 and MUC5AC secretion in MTX:MTX-rCYS×12 cocultures

When MTX:MTX-rCYS×12 cocultures were confluent, DMEM was removed, the cell layer washed with PBS, and fixed with paraformaldehyde (4% in PBS, Affymetrix). Residual aldehyde groups were inactivated by ammonium chloride (50 mM) and the cells permeabilized with saponin (0.2%, Sigma-Aldrich). Then, tissues were saturated with bovine serum albumin and saponin (1% BSA, 0.2% saponin, Sigma-Aldrich). The cells were

incubated overnight at 4°C with primary antibodies (Eu1/Eu2 directed against CYS domain, dilution 1/600; 45M1 directed against MUC5AC, dilution 1/500) in PBS 1% BSA, 0.2% saponin. Cells were washed three times with PBS before incubating with FITC–anti-mouse antibody (dilution 1/150, Santa Cruz) for 90 min at room temperature. Nuclei were counterstained with Hoechst 33258 dye (dilution 1/1000, Thermo Fisher Scientific). Observations were made using a fluorescence microscope (TCS LCSM, Leica Microsystems). To quantify cells that express rCYS×12, we performed four independent counts (mean ± SD) on a field representative of each coculture and we normalized them to 0:100 conditions. The production and secretion of rCYS×12 was confirmed by western blotting of cell lysates and culture supernatants. For western blot analysis, cells were cultivated in serum-free DMEM for 24 h. Cells were scraped and recovered in 200 μL PBS with AEBSF (1 mM), before lysis by ultrasonic treatment (VibraCell 75185, W188 probe). Supernatants were dialyzed against polyethylene glycol (100 g/L, molecular weight: 8000, Sigma-Aldrich) to concentrate them about tenfold. Samples were analyzed by SDS PAGE (8% poly-acrylamide gel) in denaturing conditions. Western blot analysis was performed using Eu1/Eu2 (dilution 1/600) or anti β-actin (dilution 1/600, Abcam) as primary antibodies. ECL was used to reveal immunoblots according to the manufacturer's instructions. Recombinant CYS×12 production was normalized to β-actin by Fiji quantification.

Rate zonal centrifugation and dot blot analysis

Supernatants from confluent MTX and MTX-rCYS×12 were fractionated by centrifugation on a 15–30% sucrose gradient using SW41Ti rotor for 90 min at 202,000 rcf, 4°C. Samples were recovered in 500 μL fractions from top to bottom and analyzed by dot blotting for the presence of MUC5AC using 45M1 antibody (dilution 1/500) or anti-CYS antibody (Eu1/Eu2, dilution 1/600), which recognizes the CYS domain of MUC5B. MUC5AC was quantified by plotting 10 μL of undiluted supernatant from each of the 5 coculture conditions on nitrocellulose membrane. The detection was carried out using 45M1 antibody (dilution 1/500) and the ECL was employed to reveal immunoblots according to the manufacturer's instructions. The semi-quantification was performed by densitometry using Fiji software.

Determination of MUC2 concentration in colonic mucus

Colonic mucus was sampled individually from WT and Tg mice housed together (n=5–8/group). The colonic tissue was opened and 150 μL of dithiothreitol (10 mM) diluted in PBS were load on the entire colonic mucosa for 2–3 min. The PBS containing reduced mucin was recovered by gentle aspiration and centrifuged to remove cellular debris and insoluble material (10,000 rcf, 5 min) and 10 μL of supernatant were plotted on nitrocellulose membrane with a dot blot apparatus. The detection of MUC2 was carried out with Mucin 2 (H-300) primary antibody (Santa Cruz, dilution 1/500). ECL was used to reveal immunoblots according to the manufacturer's instructions and the semi-quantification was performed by densitometry using Fiji software.

Online Resource 2

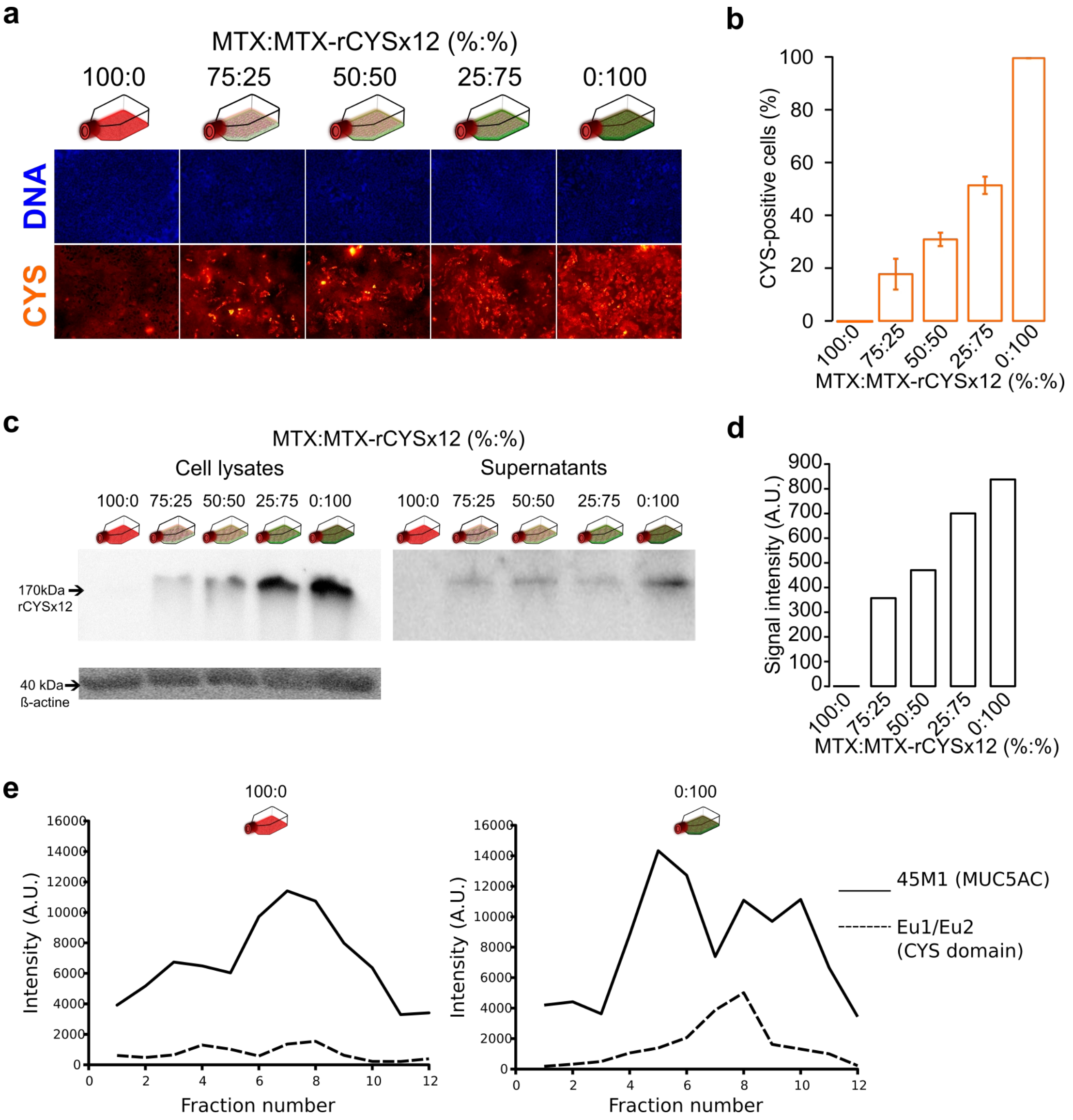

Online resource 2. Characterization of MTX:MTX-rCYS×12 cocultures.

a Immunofluorescence of MTX:MTX-rCYS×12 cocultures. rCYS×12 was observed using an antibody specifically directed against the CYS domain (red) and DNA was counterstained with Hoechst 33258 dye (blue). **b** Quantification of CYS-positive cells (mean of four independent counts ± SD). **c** Western blot experiments performed on cell lysates and supernatants from the five ratios of MTX:MTX-rCYS×12 cocultures. **d** Quantification of rCYS×12 relative expression in MTX:MTX-rCYS×12 coculture lysates normalized with β-actin. **e** Rate zonal centrifugation evidencing the association between rCYS×12 and MUC5AC.

Online Resource 3

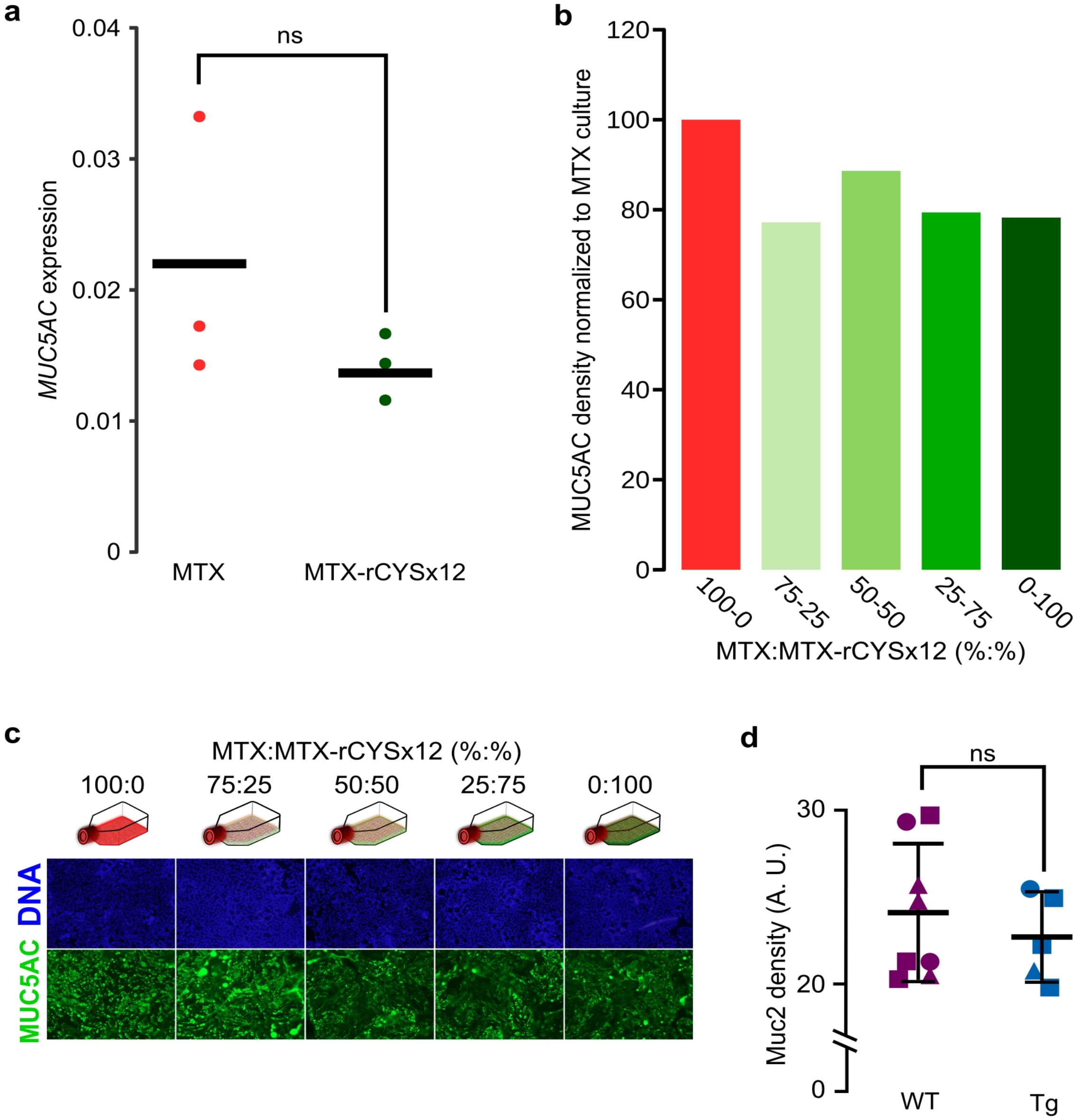

Online resource 3. Expression and production of GFM in cell coculture and in mucus from mice.

a MUC5AC expression determined by quantitative real time RT-PCR in 3 independent confluent cultures of MTX and MTX-rCYS×12 cells. ns: non significant. **b** Semi-quantification of MUC5AC production in culture supernatants from the 5 MTX:MTX-rCYS×12 cocultures determined by dot blot densitometry. **c** Immunohistochemistry showing MUC5AC production in MTX:MTX-rCYS×12 cocultures. DNA was counterstained with Hoechst 33258 dye as described in Online Resource 1. Magnification: ×20. **d** MUC2 semi-quantification in mucus recovered from colons of wild-type (WT) and transgenic (Tg) mice determined by dot blot densitometry with an anti-MUC2 antibody. ns: non significant.

Online Resource 4. *P. aeruginosa* trajectories in mucus from MTX (left) and MTX-rCYS$\times$12 (right). Movie.

Online Resource 5. *S. enterica* trajectories in scrapped mucus from WT (left) and Tg (right) mice. Movie.

Online Resource 6. Sperm cell trajectories in scrapped mucus from WT (left) and Tg (right) mice. Movie.

Online Resource 7. Characterization of beads by dynamic light scattering.

Bead	Size (nm)	ζ potential (mV)
1 μm-COOH	1034	–41.6 ± 4.1
1 μm-PEG	1259	–5.0 ± 0
200 nm-COOH	226	–46.9 ± 0
200 nm-PEG	325	–5.5 ± 0

Online Resource 8. 200 nm diameter bead diffusion in mucus from WT (left) and Tg (right) mice. Movie.

Online Resource 9. 1 μm diameter bead diffusion in mucus from WT (left) and Tg (right) mice. Movie.

Online Resource 10

Eu1/Eu2 antibody (CYS domain)

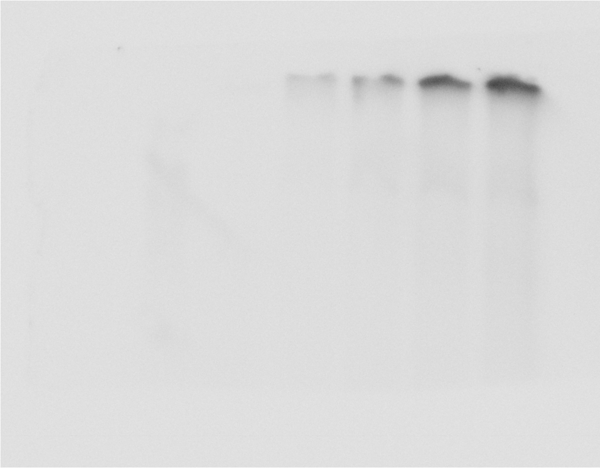

Brightfield

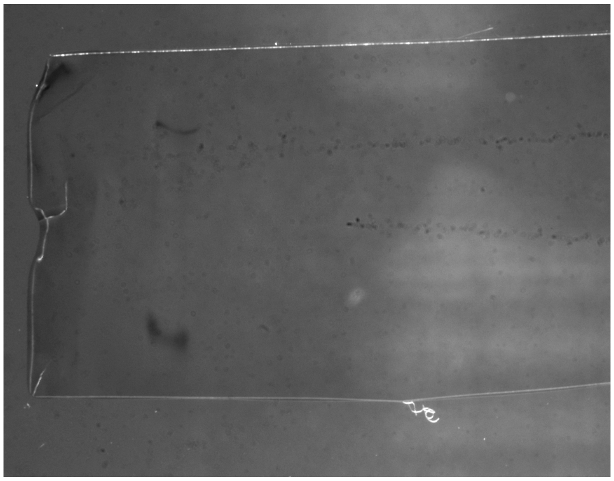

Unmodified western blot performed with an antibody directed against the CYS domain on MTX:MTX-rCYS×12 co-cultures lysates. See Online resource 2c. The full membrane is also depicted in bright field (right).

Beta-actin anti body

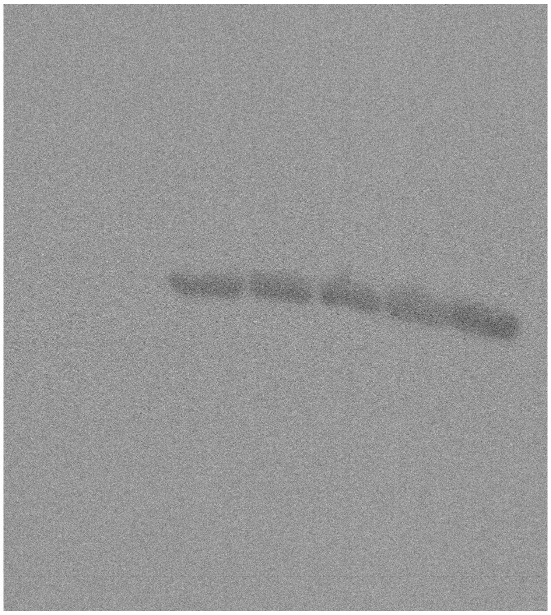

Brightfield

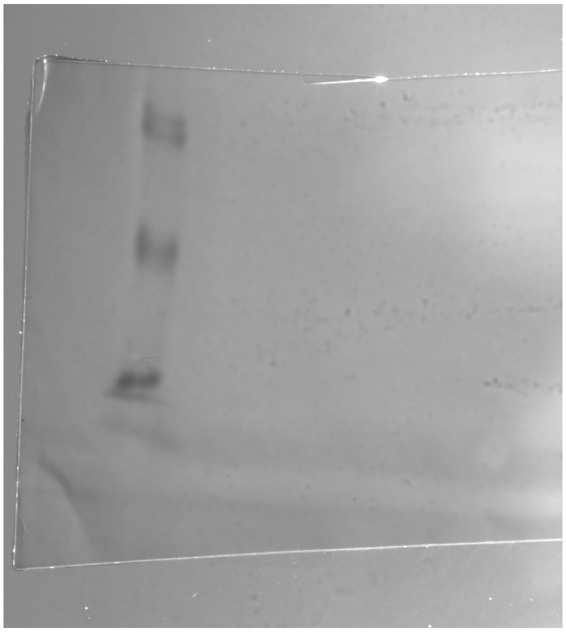

Unmodified western blot performed with an antibody directed against β-actin on MTX:MTX-rCYS×12 co-cultures lysates (left). See Online resource 2c. The full membrane is also depicted in bright field(right).
